# Clinical Characteristics and Spermatogenesis in Patients with Congenital Hypogonadotropic Hypogonadism Caused by *FGFR1* Mutations

**DOI:** 10.1155/2020/8873532

**Published:** 2020-11-28

**Authors:** Shuying Li, Yaling Zhao, Min Nie, Wanlu Ma, Xi Wang, Wen Ji, Yufan Yang, Ming Hao, Bingqing Yu, Yinjie Gao, Jiangfeng Mao, Xueyan Wu

**Affiliations:** ^1^Department of Endocrinology, Peking Union Medical College Hospital, Peking Union Medical College, Chinese Academy of Medical Sciences, Beijing 100730, China; ^2^Department of Endocrinology, The First Affiliated Hospital of Harbin Medical University, Harbin 150001, China

## Abstract

**Objective:**

The aim of this study was to investigate the clinical characteristics of patients diagnosed with congenital hypogonadotropic hypogonadism (CHH) caused by *FGFR1* (fibroblast growth factor receptor 1) gene mutations and to evaluate the effect of gonadotropin or pulsatile gonadotropin-releasing hormone (GnRH) therapy on spermatogenesis.

**Methods:**

A retrospective study was conducted on CHH patients admitted to Peking Union Medical College Hospital from January 2012 to March 2020. Clinical features and laboratory results were recorded. Testicular volume and sperm count responding to gonadotropin and pulsatile GnRH therapy were compared between the *FGFR1* mutation group and the mutation-negative group.

**Results:**

(1) *FGFR1* mutation group included 14 patients who received sperm-induction therapy, and the mutation-negative group enrolled 25 CHH patients. (2) The incidence of cryptorchidism was 50.0% (7/14) and 12.0% (3/25) in the *FGFR1* group and the mutation-negative group, respectively (*p*=0.019). The baseline testicular volume of the *FGFR1* mutation group was smaller than that of the mutation-negative group, 1.6 (0.5–2.0) mL vs. 2 (1.75–4) mL (*p*=0.033). The baseline luteinizing hormone (LH), Follicle-stimulating hormone (FSH), and testosterone levels were similar between the two groups. (3) Using the Kaplan–Meier and log-rank tests for the analysis of spermatogenesis, it was found that there was no significant difference in the first sperm appearance between the *FGFR1* mutation group and the mutation-negative group (*χ*^2^ = 1.974, *p*=0.160). The median time of spermatogenesis in the *FGFR1* mutation group was longer than that in the mutation-negative group, 16 months vs. 10 months, respectively. The cumulative spermatogenesis success rate at 12 months in the *FGFR1* mutation group (35.71%) was lower than that in the mutation-negative group (68.75%) (*p*=0.047). The sperm concentration in the mutation-negative group was more easily achieved for different thresholds compared with that in the *FGFR1* mutation group, but no significant difference was observed (*p* > 0.05) between the two groups. The last follow-up examination showed that the testicular volume was 7.00 (4.75–12.00) mL and 10.56 ± 4.82 mL (*p*=0.098), the ejaculate volume of sperm was 2.20 (1.40–2.26) mL and 3.06 ± 1.42 mL (*p*=0.175), and the sperm concentration was 7.19 (1.00–9.91) million/mL and 18.80 (4.58–53.62) million/mL (*p*=0.038) in the *FGFR1* mutation and mutation-negative groups, respectively, while the sperm motility (A%, A + B%, and A + B + C%) was similar for the two groups (*p*=0.839, 0.909, and 0.759, respectively). The testosterone level during treatment was 366.02 ± 167.03 ng/dL and 362.27 ± 212.86 ng/dL in the *FGFR1* mutation and mutation-negative groups, respectively (*p*=0.956).

**Conclusion:**

Patients with *FGFR1* mutations have a higher prevalence of cryptorchidism and smaller testicular volume. Although patients with *FGFR1* mutations have a similar rate of success for spermatogenesis compared to that of the mutation-negative patients, a longer treatment period was required and a lower sperm concentration was achieved.

## 1. Introduction

Male congenital hypogonadotropic hypogonadism (CHH) is a secondary testicular hypofunction caused by gonadotropin-releasing hormone (GnRH) secretion or action defects. The incidence in males is approximately 1/5,000–8,000 [1]. Patients are characterized by delayed pubertal development and infertility. The disease was clinically classified into Kallmann syndrome (with anosmia) and normosmic CHH. Patients typically require lifetime testosterone replacement therapy. When fertility is required, pulsatile GnRH or gonadotropin (HCG/HMG) therapy allows most patients to produce sperm.

In recent years, a number of gene mutations have been found to cause CHH, including *ANOS1 (anosmin 1), FGFR1, FGF8 (fibroblast growth factor 8), PROKR2 (prokineticin receptor 2), PROK2 (prokineticin 2), CHD7 (chromodomain helicase DNA binding protein 7), WDR11 (WD repeat domain 11), TACR3 (tachykinin receptor 3), TAC3 (tachykinin 3), KISS1R (KISS1 receptor), NSMF (NMDA receptor synaptonuclear signaling and neuronal migration factor), HS6ST1 (heparan sulfate 6-O-sulfotransferase 1), SOX2 (SRY-box transcription factor 2),* and *SEMA3A (semaphorin 3A)* [2]. Pathogenic gene mutations were identified in approximately half of the CHH patients, of which 10–20% are due to an inactivating *FGFR1* mutation [3]. Studies have shown variable degrees of function in the reproductive axis and other phenotypes are associated with different mutated genes [4].

Mutation of *FGFR1* is an important cause of CHH and previous studies have suggested that *FGFR1* mutations may cause more severe damage to the hypothalamic-pituitary-testis axis [5]. The protein encoded by *FGFR1* expressed on the cellular membranes of various cell types is a member of the fibroblast growth factor receptor (FGFR) family [6, 7]. There are three IgG-like molecules in the extracellular domain, which is the key region of ligand binding. FGFR is divided into four categories, namely, FGFR1, FGFR2, FGFR3, and FGFR4. Its ligand consists of several fibroblast growth factor (FGF) members, mainly FGF-2 and FGF-8. FGFs and the FGFR1 system play important roles in cell differentiation and proliferation, embryonic development, angiogenesis, and endocrine signaling [8]. When the ligand binds to the receptor, FGFR1 forms a homodimer, which can activate the phosphorylation of intracapsular aspartate and play a biological role through transforming growth factor-*β* (TGF-*β*) and Adenosine 5′-monophosphate (AMP) activated protein kinase (AMPK) pathways. Overactivation of FGFs and the FGFR1 system can cause multiple tumorigeneses [9], whereas inactivation mutations can cause developmental disorders of the nervous system (including olfactory bulb axons), migration disorders of GnRH neurons, and dental and skeletal development disorders [10]. *FGFR1* gene mutations are expressed in a variety of cells throughout the body and are widely involved in the proliferation and differentiation of various cell types [11].

Currently, there is insufficient research conducted on sperm-inducing therapy in patients with *FGFR1* mutations. This study aimed to evaluate the effects of spermatogenesis in patients with *FGFR1* mutations by comparing the disparity between the *FGFR1* group and the mutation-negative group.

## 2. Patients and Methods

### 2.1. Patients

CHH patients who were admitted to the Peking Union Medical College Hospital from January 2012 to March 2020 were recruited, and the clinical data were retrospectively collected.

Inclusion criteria: (1) CHH males were diagnosed if the patient fulfilled all the following conditions: no pubertal development after 18 years old, blood testosterone ≤100 ng/dL with a low or inappropriately normal level of serum gonadotropins, normal secretion of other anterior pituitary hormones, and normal magnetic resonance (MR) imaging in the seller region; (2) sperm-inducing therapy was administered to patients; (3) *FGFR1* mutation group (*FM* group): *FGFR1* gene mutations were identified by high-throughput next-generation sequencing, then verified by Sanger sequencing, and the pathogenicity was determined by the American College of Medical Genetics and Genomics (ACMG) guidelines [12]; (4) the control group (mutation-negative group, MN group): CHH patients who had no gene mutation detected after high-throughput next-generation sequencing.

**Figure 1:**
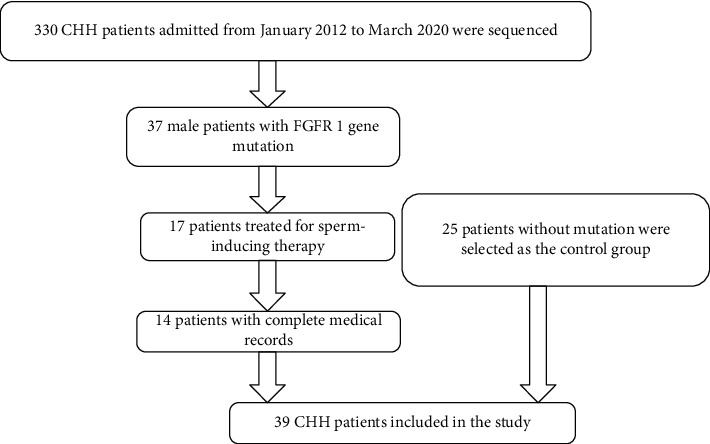
Flow chart of patient inclusion criteria.

Exclusion criteria: (1) patients with any other gene mutations that were pathogenic or likely pathogenic genes of CHH by the ACMG guidelines; (2) patients with uncompleted medical records; (3) patients who did not receive sperm-inducing treatment. Written informed consent was obtained from all patients. The patient inclusive procedure is shown in Figure 1.

### 2.2. Therapeutic Regimes and Patient Follow-Up

Pathogenic genes were screened by a next-generation sequencing panel, which included (but not limited to) *FGF8* and its receptor *FGFR1*, *GNRH1* and its receptor *GNRHR*, *PROK2* and its receptor *PROKR2*, *TAC3* and its receptor *TACR3*, *LEP* and its receptor *LEPR*, and *CHD7*, *ANOS1*, *KISS1R*, *NSMF*, *WDR11*, *LHB*, *FSHB,* and *PCSK1* [1, 13–16]. The CHH-related genes included in the panel are shown in Supplementary Table 1. High-throughput next-generation sequencing was used to identify patients with *FGFR1* mutations. Sanger sequencing was performed on patients and their parents to verify and track *FGFR1* mutations (The specific process and method of the next-generation sequencing are shown in Supplementary Material 2).

Clinical data collected from *FGFR1* gene mutation and MN patients undergoing spermatogenesis treatment included the following information: age; body mass index (BMI); clinical characteristics outside the gonadal axis; age for starting spermatogenesis treatment; a history of cryptorchidism; testis volume before and after spermatogenesis treatment; a history of testosterone replacement therapy before spermatogenesis treatment; the ejaculate volume and concentration in the follow-up duration; sperm motility, which was classified as fast progressive sperm (A), slow progressive sperm (B), and nonprogressive sperm (C); LH, FSH, and testosterone levels during the treatment. For LH, FSH, and testosterone, the average values of the last two visits were taken for data analysis. The ejaculate volume, sperm concentration, and sperm motility at the last follow-up were analyzed.

Testicular volume was measured by two gonadal clinical specialists. During the follow-up examination, testis volume was measured by direct comparison with the orchidometer. If there were bilateral testicles, the average value was taken. If the testis was unilateral (cryptorchidism was on the other side), the descended testis volume was taken for statistical analysis. The average testicular size of the last two visits was defined as the testicular size after therapy for data analysis. The first sperm was detected under the microscope, and semen was centrifugated if necessary.

Patients discontinued androgen therapy (if used) for at least 3 months before starting gonadotropins therapy. Human chorionic gonadotrophin/human menopausal gonadotropin (HCG/HMG) treatment included intramuscular injection of 2000–3000 IU of HCG and 75 IU of HMG twice a week. The HCG dose was adjusted according to the testosterone level, which was maintained at 200–400 ng/dL. Pulsatile GnRH treatment included a subcutaneous injection of pulsatile gonadorelin, starting at a dosage of 10 *μ*g/90 min. The dosage was adjusted according to the levels of LH and testosterone, and the level of testosterone was maintained at 200–400 ng/dL. Follow-up was conducted at an interval of 3–6 months. The seminal test, sex hormone, and testis volume were measured during each visit.

### 2.3. Statistical Analysis

SPSS version 23.0 was used for analyzing the difference between CHH patients with and without *FGFR1* gene mutations who received spermatogenic therapy. Normally distributed data are expressed as the mean ± SD, and nonnormally distributed data are expressed as the median (quartiles). The nonpaired *t*-test was used to compare data between two groups, such as the plasma testosterone and testicular volumes, if the data were normally distributed. If the two groups of data were not normally distributed, nonparametric tests were used to test the significance of the difference between the two groups. The Kaplan–Meier and log-rank tests were used for the analysis of spermatogenesis. The chi-square test was used to compare the rates between groups. When *p* < 0.05, it was considered statistically significant.

## 3. Results

### 3.1. Baseline Characteristic Comparison between the FGFR1 Group and the Mutation-Negative Group

From January 2012 to March 2020, 330 CHH patients underwent panel gene sequencing. Among them, 37 patients had *FGFR1* gene mutation. 17 among the 37 patients received sperm-inducing treatment. Among the 17 patients, 14 had complete medical records. A total of 14 patients with *FGFR1* gene mutations were included. 25 patients without mutations who matched the *FGFR1* group with a complete medical record and matched the age of spermatogenesis, preuse, and use time of testosterone were selected as the control group (Figure 1). The mutation types of *FGFR1* were heterogeneous, including 11 missense mutations, 2 frame shift mutations, and 1 deletion mutation. Four patients with *FGFR1* gene mutations were sequenced for the source of the mutations, and all were de novo mutations (Table 1).

In the FM group, 11 patients had previous testosterone therapy for 1 (1–2) years, while in the MN group, 12 patients had been treated with testosterone for 1 (0.5–4) years. The age for starting spermatogenic treatment was 20.00 (18.75–24.75) years and 24.12 ± 5.75 years (*p*=0.297), the testicular volume was 1.6 (0.5–2.0) mL and 2.0 (1.75–4) mL (*p*=0.033), the LH level was 0.20 (0.10–0.70) IU/L and 0.35 (0.20–0.72) IU/L, and the baseline testosterone was 24.1 (18.58–37.25) ng/dL and 32.70 (20.65–46.5) ng/dL (measured after stopping testosterone replacement therapy for at least 3 months) in the FM group and the MN group, respectively (Table 2).

### 3.2. *FGFR1* Mutation Group Had Higher Incidence of Cryptorchidism

There were seven cases of cryptorchidism in the *FGFR1* group (50.0%): three were unilateral and four were bilateral. There were three cases of cryptorchidism in the MN group (12.0%): one was unilateral and two were bilateral (Table 2).

### 3.3. Treatment Time for the First Appearance of Spermatozoa

In the MN group, one patient had received sperm-inducing treatment, but there was no information from the first follow-up. Therefore, this patient was excluded from the spermatogenesis analysis. The mean follow-up period was similar between the two groups: 26.36 ± 8.54 months in the FM group and 22.54 ± 8.74 months in the MN group (*p*=0.199) (Table 3).

**Figure 2:**
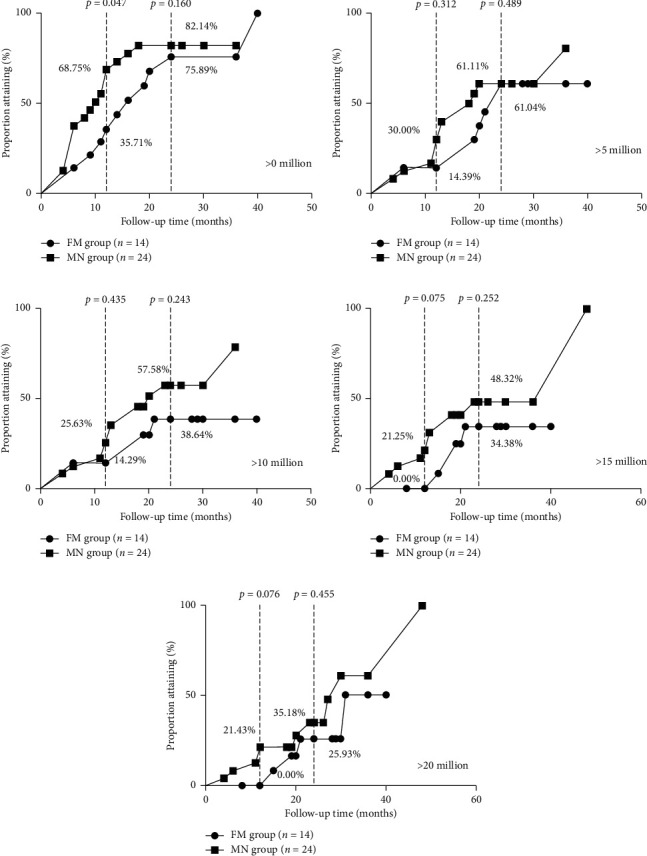
(a) Kaplan–Meier curve for achieving sperm concentration more than 0 million/ml (first sperm appearance) in FM and MN groups. (b) Kaplan–Meier curve for achieving sperm concentration more than 5 million/ml (first sperm appearance) in FM and MN groups. (c) Kaplan–Meier curve for achieving sperm concentration more than 10 million/ml (first sperm appearance) in FM and MN groups. (d) Kaplan–Meier curve for achieving sperm concentration more than 15 million/ml (first sperm appearance) in FM and MN groups. (e) Kaplan–Meier curve for achieving sperm concentration more than 20 million/ml (first sperm appearance) in FM and MN groups.

The median time for the first sperm appearance in semen of the FM group was longer than that of the MN group (16 vs. 10 months, 95% CI: 7.62 to 24.39 months and 5.45 to 14.55 months) after induced spermatogenesis therapy. Analysis of spermatogenesis using the Kaplan–Meier and log-rank test showed that there was no difference in the overall spermatogenesis rate between the two groups (*χ*^2^ = 1.974, *p*=0.160) (Figure 2(a)). The successful rate of spermatogenesis at 12 and 24 months of treatment was 35.71% and 75.89% in the FM group, and 68.75% and 82.14% in the MN group, with *p* values of 0.047 and 0.160 between the two groups, respectively (Table 3 and Figure 2(a)). At the final visit, the testis volume was 7.00 (4.75–12.00) and 10.56 ± 4.82 mL (*p*=0.098), and the testosterone level was 366.02 ± 167.03 and 362.27 ± 212.86 (*n* = 24) ng/dL (*p*=0.956) in the FM and MN groups, respectively (Table 3).

In the FM group, four patients were treated with pulsatile GnRH, and the final dosage of GnRH was gradually adjusted to 9.5 ± 3.42 (6–14) *μ*g/90 min, the LH level increased to 8.46 ± 3.31 IU/L, the FSH level increased to 9.77 ± 6.20 IU/L, and the testosterone level increased to 286.95 ± 130.11 ng/dL. Among these patients, one switched from 12-month gonadotropin therapy to pulsatile GnRH therapy. Ten patients in the *FM* group received gonadotropin therapy. The final adjusted dosage was 2600 ± 516.40 (2000–3000) IU for HCG and 75 IU for HMG, twice weekly. The testosterone level increased to 397.65 ± 189.78 ng/dL (Table 3).

In the MN group, 13 patients received pulsatile GnRH treatment. The LH level increased to 7.59 ± 4.07 IU/L, the FSH level increased to 9.75 ± 4.01 IU/L, the testosterone level increased to 304.54 ± 167.03 ng/dL, and the final dosage of GnRH was gradually adjusted to 10.08 ± 4.11 (4–20) *µ*g/90 min. The other 11 patients received gonadotropin treatment and the testosterone level increased to 431.76 ± 247.42 ng/dL. The final adjusted dosage was 2500 ± 527.05 (2000–3000) IU for HCG and 75 IU for HMG, twice weekly (Table 3).

### 3.4. Semen Parameters after Sperm-Inducing Treatment

**Figure 3:**
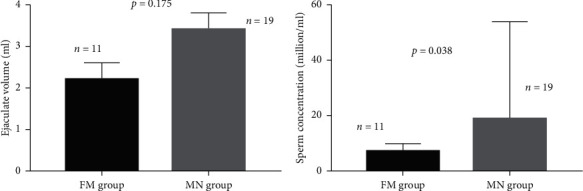
Sperm parameters (ejaculate volume and concentration) after spermatogenesis treatment (the plot was described as median with interquartile range).

Analysis of attaining different sperm concentration thresholds (0, 5, 10, 15, and 20 million/mL) using Kaplan–Meier and log-rank tests showed that there was no significant difference in overall spermatogenesis rate between the FM and MN groups (Table 4 and Figure 2). The median time for sperm concentration to achieve 5 million/mL was 24 and 18.5 months in the FM and MN groups, respectively. The median time for sperm concertation to achieve 10 million/mL in the MN group was 20 months. However, other median times for sperm concentration to achieve different thresholds in the two groups cannot be obtained due to the small size of samples. During the last visit, the ejaculate volume in the FM and MN groups was measured to be 2.20 (1.40–2.26) mL and 3.06 ± 1.42 mL (*p*=0.175), and the sperm concentration was 7.19 (1.00–9.91) million/mL and 18.80 (4.58–53.62) million/mL, respectively (*p*=0.038) (Figure 3), while the sperm motility (A%, A + B%, and A + B + C%) was similar between the two groups (*p*=0.839, 0.909, and 0.759, respectively).

### 3.5. Patients Who Failed to Produce Sperm after Two Years of Treatment

Six patients failed to produce sperm after two years of treatment, two were in the FM group and four were in the MN group. Among these patients, three had a history of cryptorchidism. During the follow-up examination, the testosterone mean level was as low as 79.65 ng/dL (32.8–148 ng/dL), and four patients had testis size smaller than or equal to 5 mL (Table 5).

## 4. Discussion

Our research focused on the clinical characteristics and spermatogenesis of patients with *FGFR1* gene mutations who underwent sperm-inducing treatment. We found that the *FM* patients had a higher incidence of cryptorchidism (50.0% vs. 12.0%, *p*=0.019) and smaller testis size (1.6 vs. 2.0 mL, *p*=0.033) compared to patients without positive gene mutations. After a year of sperm promoting therapy, the MN group had a higher rate of achieving successful spermatogenesis than the FM group (35.71% vs. 68.75%, *p*=0.047). After two years of therapy, 75.9% of the patients in the FM group achieved successful spermatogenesis, similar to that of patients without gene mutations (82.14%). The median time needed for first sperm achievement in the FM gene group was longer compared to that of the MN group (16 months vs. 10 months). At the last follow-up, the sperm concentration in the FM group was lower than that in the MN group (7.19 million/mL vs. 18.80 million/mL), although the sperm mobility was similar for the two groups.

In our study, mutated *FGFR1* was speculated as pathogenic and disease-causing, based on the following evidence: (1) in this study, patients in the FM group were single-gene mutant; (2) according to the criteria of the ACMG guidelines [12], half of the mutants were pathogenic or likely pathogenic (7/14); (3) four patients were further analyzed and had de novo mutations. Our previous study showed that most of the *FGFR1* gene mutations (11/12) were de novo (Supplementary Table 2), suggesting that the mutated *FGFR1* is most likely pathogenic in this group.

Patients with *FGFR1* mutations have a more severe impairment of the reproductive phenotype, mainly manifested by a high incidence of cryptorchidism and smaller testis volume during spermatogenesis treatment. Our study found that the incidence of cryptorchidism in patients with *FGFR1* gene mutations was 50%, which is consistent with previous studies [5]. The prevalence of cryptorchidism in the CHH population is 10–20% [13, 17]. The incidence of cryptorchidism in patients with *FGFR1, ANOS1,* and *CHD7* mutations is 50–60% [5, 18], 38.1–66% [19, 0], and 50–70% [21], respectively. As *FGFR1* is expressed in Leydig cells, Sertoli cells, and spermatogonia, its function is important for testicular development and spermatogenesis [22–26]. Therefore, the inactivating mutation may impair spermatogenesis through a testicular pathway. Currently, there has not been a study focusing on sperm production in patients with *FGFR1* gene mutations.

The presence of cryptorchidism is not only a reflection of severe reproductive axis damage but also a harmful predictive factor of spermatogenesis [27, 28]. It has been shown that the success rate of spermatogenesis in CHH with cryptorchidism is lower than patients without cryptorchidism (50% vs. 67%, *p*=0.032). In these patients, a longer time was needed to achieve the first appearance of sperm [29]. Several detrimental conditions may impair the progress of spermatogenesis. First, undescended testis was associated with a higher surrounding temperature and the optimal temperature for spermatogenesis (33°C) cannot be maintained [30]. Higher ambient temperature would impair gonocyte maturation [31]. Secondly, additional oxidative stress and inflammation in the undescended testis would impair sperm quality [32]. The higher incidence of cryptorchidism may be the reason for the longer time of spermatogenesis in the FM group.

In our study, after two years of sperm-inducing treatment, the success rate of the FM and MN groups was 75.89% and 82.14%, respectively. According to the meta-analysis results from several studies, the overall success rate is 74–90% in patients receiving pulsatile GnRH therapy [33] and 69.2–94.4% in patients receiving gonadotropin therapy [34, 35]. No clear genetic diagnosis was made in these CHH patients. Although the success rate of spermatogenesis in *FGFR1* and MN groups was similar, the median time for first sperm appearance was longer in the FM group.

There is no study on the evaluation of spermatogenic effects on patients with different genotypes. The possible reasons are as follows: (1) it is difficult to make a gene diagnosis on patients with CHH, as many patients do not have gene variations, while some have multiple gene mutations [36]. (2) Patients with the same mutation have different clinical phenotypes. For example, for the same *FGFR1* mutation, some presented CHH, while others showed delayed puberty [1, 37]. Patients with the same *PROKR2* mutations also have variable reproductive conditions [5]. These variable conditions make it difficult to accurately evaluate the effects of sperm promoting therapy. (3) The number of patients with the same gene mutation and spermatogenic treatment is too small to be statistically analyzed.

*FGFR1* may impair spermatogenesis at the hypothalamus, pituitary, and testis levels. It was found that 25.6% (23/90) of CHH patients had a poor testicular response to gonadotropins and 11.1% (10/90) had a poor response to pulsatile GnRH therapy, indicating defects in the pituitary gland and testicular levels [38]. *FGFR1* gene mutations may result in a weaker response of Leydig cells and spermatogonia to gonadotropins. Therefore, patients need longer treatment time to promote testicular volume and spermatogenesis.

In this study, two patients with *FGFR1* gene mutations and cryptorchidism history had a failure of spermatogenesis after two years of treatment. In patient 1 (Table 4), the LH and FSH level increased during pulsatile GnRH therapy, while the testosterone level did not significantly increase. In patient 2, the testosterone level did not significantly increase after gonadotropin therapy. Both patients indicated impaired Leydig cell function. In the MN group, four patients had a failure of spermatogenesis after two years of treatment. All of the patients did not reach normal levels of testosterone, suggesting that patients in the MN group may also have damaged Leydig cell function.

The classical pathogenesis of CHH is associated with defective GnRH neuron differentiation and migration. *FGFR1* gene mutations not only cause defective GnRH neurons in the hypothalamus but also lead to the damage of pituitary gonadotropin cells. Some mutations may result in anterior pituitary hypoplasia and a deficiency of multiple pituitary hormones [39]. The multilevel damage in the gonadal axis may also explain why the FM group requires longer time for sperm production.

The shortcomings of this study are as follows: (1) the sample size is too small to accurately reflect the entire possible spermatogenic outcomes in the *FGFR1* gene mutation population. However, as a rare disease, the description of 14 cases provides a valuable opportunity to observe the change of reproductive axis function. (2) In this study, the MN group was defined by having no known CHH related genes. Other gene mutations causing CHH could not be excluded. (3) Pulsatile GnRH and gonadotropin therapies have different mechanisms to induce sperm. Due to the small sample size, the disparity between the two therapeutic methods could not be further analyzed. (4) The mutation pathogenicity was determined based on the ACMG guidelines, not by in vitro verification.

In conclusion, gonadal axis defects were more severe in CHH patients with *FGFR1* mutations, presenting a higher incidence of cryptorchidism, smaller testicular size, and longer time required for sperm production. However, the rate of successful spermatogenesis in different sperm concentration thresholds was similar between patients with *FGFR1* mutations and nonmutation patients, providing valuable evidence and confidence for sperm promoting therapy for these patients.

## Figures and Tables

**Table 1:** *FGFR1* mutation type, specific site information, and pathogenicity.

Mutation types	Mutation site	Inherited	Pathogenicity
Missense mutation	p.V184M (c.550G > A)	Unknown	Likely pathogenic
	p.R477T (c.1430G > C)	Unknown	Uncertain
	p.A342V (c.1025C > T)	Unknown	Uncertain
	p.F186I (c.556T > A)	Unknown	Uncertain
	p.H452P (c.1355A > C)	Unknown	Uncertain
	p.V184M (c.550G > A)	Unknown	Likely pathogenic
	p.T340M (c.1019C > T)	Unknown	Likely pathogenic
	p.G260R (c.778G > C)	De novo	Likely pathogenic
	p.R254W (c.760C > T)	De novo	Likely pathogenic
	p.R424H (c.1271G > A)	Unknown	Uncertain
	p.R165Q (c.494G > A)	Unknown	Uncertain

Frame shift mutation	p.L188Hfs*∗*5 (c.562_563insAC)	De novo	Pathogenic
	p.Y280Lfs*∗*2 (c.838dupT)	De novo	Pathogenic

Deletion mutation	p.733_733del (c.2197_2199delATG)	Unknown	Uncertain

**Table 2:** Comparison of clinical characteristics between the *FGFR1* gene group and the mutation-negative group at baseline.

Groups	*FGFR1* mutation (*n* = 14)	Mutation-negative (*n* = 25)	*p*
Kallmann/nCHH (n/*n*)	8/6	20/5	0.156
Gonadotropin/GnRH pulsatile (n/*n*)	10/4	12/13	0.193
Number of testosterone users before sperm promoting therapy (*n*, %)	11, 78.57%	12, 480.00%	0.093
Previous testosterone treatment course (years)	1 (1–2) (*n* = 11)	1 (0.5–4) (*n* = 12)	0.441
Cryptorchidism or history of cryptorchidism (*n*, %)	7, 50.0% (Unilateral/Bilateral = 3/4)	3, 12.0%*∗* (Unilateral/Bilateral = 1/2)	0.019
Age of start for spermatogenic treatment (years)	20.00 (18.75–24.75)	24.12 ± 5.75	0.297
Baseline testicular size (mL)	1.6 (0.5–2.0)	2.0 (1.75–4)*∗∗*	0.033
Baseline LH (IU/L)	0.20 (0.10–0.70)	0.35 (0.20–0.72)	0.318
Baseline FSH (IU/L)	1.01 ± 0.85	0.60 (0.22–1.58)	0.714
Baseline testosterone (ng/dL)	24.1 (18.58–37.25)	32.70 (20.65–46.5)	0.198
LH 60 min after triptorelin stimulating test (IU/L)	2.3 (1.16–5.68)	1.75 (0.77–5.78)	0.529
Nonreproductive phenotype	Obesity (*n* = 2)	Obesity (*n* = 3); diabetes (*n* = 1); solitary kidney (*n* = 1); strabismus (*n* = 1); unilateral ptosis (*n* = 1); multiple facial nevi (*n* = 1);	

**Table 3:** Sperm production during therapy.

Groups	FGFR1 mutation (*n* = 14)	Mutation-negative (*n* = 24)	*p*
Mean follow-up time (months)	26.36 ± 8.54	22.54 ± 8.74	0.199
Cumulative success rate of achieving first sperm appearance in 1 year	35.71%	68.75%	0.047
Cumulative success rate of achieving first sperm appearance in 2 years	75.89%	82.14%	0.160
Testicular volume (mL)	7.00 (4.75–12.00) (*n* = 14)	10.56 ± 4.82 (*n* = 24)	0.098
Testosterone (ng/dL)	366.02 ± 167.03 (*n* = 14)	362.27 ± 212.86 (*n* = 24)	0.956
Testosterone in pulsatile GnRH therapy (ng/dL)	286.95 ± 130.11 (*n* = 4)	304.54 ± 167.03 (*n* = 13)	0.857
Testosterone in gonadotropin therapy (ng/dL)	397.65 ± 189.78 (*n* = 10)	431.76 ± 247.42 (*n* = 11)	0.729
FSH in GnRH pulsatile therapy (IU/L)	9.77 ± 6.20 (*n* = 4)	9.75 ± 4.01 (*n* = 13)	0.993
LH in GnRH pulsatile therapy (IU/L)	8.46 ± 3.31 (*n* = 4)	7.59 ± 4.07 (*n* = 13)	0.737

**Table 4:** Semen parameters after sperm-inducing treatment.

Groups		FGFR1 mutation (*n* = 14)	Mutation-negative (*n* = 24)	*p*
Cumulative rate of sperm concentration >5 million	1 years	14.39%	30.00%	0.312
	2 years	61.04%	61.11%	0.489
Cumulative rate of sperm concentration >10 million	1 years	14.29%	25.63%	0.435
	2 years	38.64%	57.58%	0.243
Cumulative rate of sperm concentration >15 million	1 years	0.00%	21.25%	0.075
	2 years	34.38%	48.32%	0.252
Cumulative rate of sperm concentration >20 million	1 years	0.00%	21.43%	0.076
	2 years	25.93%	35.18%	0.455
Ejaculate volume (mL)		2.20 (1.40–2.26) (*n* = 11)	3.06 ± 1.42 (*n* = 19)	0.175
Sperm concentration (million/mL)		7.19 (1.00–9.91) (*n* = 11)	18.80 (4.58–53.62) (*n* = 19)	0.038
Fast progressive sperm (A) (%)		18.00 ± 19.92 (*n* = 11)	16.77 ± 13.00 (*n* = 19)	0.839
Sperm progressive motility (A + B) (%)		33.83 ± 26.31 (*n* = 11)	34.88 ± 22.71 (*n* = 19)	0.909
Sperm total mobility (A + B + C) (%)		37.26 ± 29.21 (*n* = 11)	40.38 ± 24.91 (*n* = 19)	0.759

Sperm concentration, sperm volume, fast progressive sperm, sperm progressive motility, and sperm total mobility were obtained from the last visit.

**Table 5:** Clinical manifestations and hormone levels in patients who had a failure of spermatogenesis.

Failure cases	Diagnosis	Gene mutation type	General condition before spermatogenic treatment		Therapeutic regime	Follow-up	
Patient 1	nCHH	*FGFR1*	Age for starting spermatogenic therapy (year)	20	GnRH pulsatile	Follow-up duration (months)	36
		p.V184M (GTG->ATG)	BMI (kg/m^2^)	32.8		Testicular size (mL)	5/5
			History of cryptorchidism or cryptorchidism (yes/no)	Yes		LH (IU/L)	3.88
			Testicular size (mL)	2/2		FSH (IU/L)	3.8
			LH (IU/L)	0.12		Testosterone (ng/dL)	78.6
			FSH (IU/L)	0.27			
			LH 60 min (IU/L)	1.05			
			Testosterone (ng/dL)	31			

Patient 2	Kallmann syn	*FGFR1*	Age for starting spermatogenic therapy (year)	19	Gonadotropin	Follow-up duration (months)	24
		p.F186I (TTC->ATC)	BMI (kg/m^2^)	3.8		Testicular size (mL)	1/1
			History of cryptorchidism or cryptorchidism (yes/no)	19.4		LH (IU/L)	0
			Testicular size (mL)	Yes		FSH (IU/L)	0.15
			LH (IU/L)	0/ 1		Testosterone (ng/dL)	37.5
			FSH (IU/L)	0.13			
			LH 60 min (IU/L)	0.6			
			Testosterone (ng/dL)	1.01			

Patient 3	Kallmann syn	Mutation-negative	Age for starting spermatogenic therapy (year)	28	GnRH pulsatile	Follow-up duration (months)	24
			BMI (kg/m^2^)	37.4		Testicular size (mL)	3/3
			History of cryptorchidism or cryptorchidism (yes/no)	No		LH (IU/L)	2.4
			Testicular size (mL)	3/3		FSH (IU/L)	4.6
			LH (IU/L)	0.86		Testosterone (ng/dL)	71
			FSH (IU/L)	0.86			
			LH 60 min (IU/L)	1.30			
			Testosterone (ng/dL)	67.00			

Patient 4	Kallmann syn	Mutation-negative	Age for starting spermatogenic therapy (year)	19	GnRH pulsatile	Follow-up duration (months)	36
			BMI (kg/m^2^)	24.6		Testicular size (mL)	10/8
			History of cryptorchidism or cryptorchidism (yes/no)	Yes		LH (IU/L)	7
			Testicular size (mL)	0/0		FSH (IU/L)	3.5
			LH (IU/L)	0.3		Testosterone (ng/dL)	32.8
			FSH (IU/L)	0.43			
			LH 60 min (IU/L)	1.06			
			Testosterone (ng/dL)	30.00			

Patient 5	Kallmann syn	Mutation-negative	Age for starting spermatogenic therapy (year)	25	Gonadotropin	Follow-up duration (months)	30
			BMI (kg/m^2^)	24.2		Testicular size (mL)	3/3
			History of cryptorchidism or cryptorchidism (yes/no)	No		LH (IU/L)	0.20
			Testicular size (mL)	1/1		FSH (IU/L)	0.18
			LH (IU/L)	0.35		Testosterone (ng/dL)	110
			FSH (IU/L)	0.35			
			LH 60 min (IU/L)	0.57			
			Testosterone (ng/dL)	20.5			

Patient 6	nCHH	Mutation-negative	Age for starting spermatogenic therapy (year)	24	GnRH pulsatile	Follow-up duration (months)	26
			BMI (kg/m^2^)	24.1		Testicular size (mL)	12/12
			History of cryptorchidism or cryptorchidism (yes/no)	No		LH (IU/L)	9.04
			Testicular size (mL)	2.5/2.5		FSH (IU/L)	4.99
			LH (IU/L)	0.2		Testosterone (ng/dL)	148
			FSH (IU/L)	0.2			
			LH 60 min (IU/L)	1.04			
			Testosterone (ng/dL)	34			

Testicular volume, FSH, LH, and testosterone levels were all obtained by the average of the last two measurements.

## Data Availability

The data used to support the findings of this study are included within the article.

## Supplementary Materials

Supplementary Materials Supplementary material 1 includes two supplementary tables: supplementary table 1 shows CHH-related genes included in the panel; supplementary table 2 shows the source analysis of *FGFR1, PROKR2, ANOS1* gene mutations. Supplementary material 2 shows gene sequencing flow.

## References

[1] Topaloglu A. K., Kotan L. D. (2016). Genetics of hypogonadotropic hypogonadism. *Endocrine Development*.

[2] Maione L., Dwyer A. A., Francou B. (2018). Genetics in endocrinology: genetic counseling for congenital hypogonadotropic hypogonadism and kallmann syndrome: new challenges in the era of oligogenism and next-generation sequencing. *European Journal of Endocrinology*.

[3] Amato L. G. L., Montenegro L. R., Lerario A. M. (2019). New genetic findings in a large cohort of congenital hypogonadotropic hypogonadism. *European Journal of Endocrinology*.

[4] Stamou M. I., Georgopoulos N. A. (2018). Kallmann syndrome: phenotype and genotype of hypogonadotropic hypogonadism. *Metabolism*.

[5] Mao J. (2012). *Influence of Gene Mutations on Hypothalamus Pituitary Gonad Axis Function and Gonadotropin Induced Spermatogenesis in Male Patients with Idiopathic Hypogonadotropic Hypogonadism*.

[6] Koika V., Varnavas P., Valavani H. (2013). Comparative functional analysis of two fibroblast growth factor receptor 1 (FGFR1) mutations affecting the same residue (R254W and R254Q) in isolated hypogonadotropic hypogonadism (IHH). *Gene*.

[7] Kurowski A., Molotkov A., Soriano P. (2019). FGFR1 regulates trophectoderm development and facilitates blastocyst implantation. *Developmental Biology*.

[8] Gonçalves C., Bastos M., Pignatelli D. (2015). Novel FGFR1 mutations in Kallmann syndrome and normosmic idiopathic hypogonadotropic hypogonadism: evidence for the involvement of an alternatively spliced isoform. *Fertility and Sterility*.

[9] Katoh M. (2016). FGFR inhibitors: effects on cancer cells, tumor microenvironment and whole-body homeostasis (Review). *International Journal of Molecular Medicine*.

[10] Woodbury M. E., Ikezu T. (2014). Fibroblast growth factor-2 signaling in neurogenesis and neurodegeneration. *Journal of Neuroimmune Pharmacology*.

[11] Simonis N., Migeotte I., Lambert N. (2013). FGFR1mutations cause Hartsfield syndrome, the unique association of holoprosencephaly and ectrodactyly. *Journal of Medical Genetics*.

[12] Biesecker L. G., Harrison S. M. (2018). The ACMG/AMP reputable source criteria for the interpretation of sequence variants. *Genetics in Medicine*.

[13] Bhagavath B., Podolsky R. H., Ozata M. (2006). Clinical and molecular characterization of a large sample of patients with hypogonadotropic hypogonadism. *Fertility and Sterility*.

[14] Topaloğlu A. K. (2018). Update on the genetics of idiopathic hypogonadotropic hypogonadism. *Journal of Clinical Research in Pediatric Endocrinology*.

[15] Kim Y. J., Osborn D. P., Lee J. Y. (2018). WDR11-mediated Hedgehog signalling defects underlie a new ciliopathy related to Kallmann syndrome. *EMBO Reports*.

[16] Semple R. K., Topaloglu A. K. (2010). The recent genetics of hypogonadotrophic hypogonadism - novel insights and new questions. *Clinical Endocrinology*.

[17] Liu P. Y., Baker H. W. G., Jayadev V., Zacharin M., Conway A. J., Handelsman D. J. (2009). Induction of spermatogenesis and fertility during gonadotropin treatment of gonadotropin-deficient infertile men: predictors of fertility outcome. *The Journal of Clinical Endocrinology & Metabolism*.

[18] Pitteloud N., Meysing A., Quinton R. (2006). Mutations in fibroblast growth factor receptor 1 cause Kallmann syndrome with a wide spectrum of reproductive phenotypes. *Molecular and Cellular Endocrinology*.

[19] Nie M., Xu H., Chen R. (2017). Analysis of genetic and clinical characteristics of a Chinese Kallmann syndrome cohort with ANOS1 mutations. *European Journal of Endocrinology*.

[20] Salenave S., Chanson P., Bry H. (2008). Kallmann’s syndrome: a comparison of the reproductive phenotypes in men carrying KAL1 and FGFR1/KAL2 mutations. *The Journal of Clinical Endocrinology & Metabolism*.

[21] Hsu P., Ma A., Wilson M. (2014). Charge syndrome: a review. *Journal of Paediatrics and Child Health*.

[22] Hu Y., Bouloux P.-M. (2010). Novel insights in FGFR1 regulation: lessons from Kallmann syndrome. *Trends in Endocrinology & Metabolism*.

[23] Hadziselimovic N. O., de Geyter C., Demougin P., Oakeley E. J., Hadziselimovic F. (2010). Decreased expression of FGFR1, SOS1, RAF1 genes in cryptorchidism. *Urologia Internationalis*.

[24] Le Magueresse-Battistoni B., Wolff J., Morera A. M., Benahmed M. (1994). Fibroblast growth factor receptor type 1 expression during rat testicular development and its regulation in cultured Sertoli cells. *Endocrinology*.

[25] Chen L., Li X., Wang Y. (2019). Fibroblast growth factor 1 promotes rat stem Leydig cell development. *Front Endocrinol (Lausanne)*.

[26] Cancilla B., Risbridger G. P. (1998). Differential localization of fibroblast growth factor receptor−i, −2, −3, and −4 in fetal, immature, and adult rat Testes1. *Biology of Reproduction*.

[27] Finkel D. M., Phillips J. L., Snyder P. J. (1985). Stimulation of spermatogenesis by gonadotropins in men with hypogonadotropic hypogonadism. *New England Journal of Medicine*.

[28] Kirk J. M. W., Savage M. O., Grant D. B., Bouloux P.-M. G., Besser G. M. (1994). Gonadal function and response to human chorionic and menopausal gonadotrophin therapy in male patients with idiopathic hypogonadotrophic hypogonadism. *Clinical Endocrinology*.

[29] Liu Z., Mao J., Xu H. (2019). Gonadotropin-induced spermatogenesis in CHH patients with cryptorchidism. *International Journal of Endocrinology*.

[30] Cobellis G., Noviello C., Nino F. (2014). Spermatogenesis and cryptorchidism. *Front Endocrinol (Lausanne)*.

[31] Ivell R., Hartung S. (2003). The molecular basis of cryptorchidism. *Molecular Human Reproduction*.

[32] Imamoglu M., Bulbul S. S., Kaklikkaya N., Sarihan H. (2012). Oxidative, inflammatory and immunologic status in children with undescended testes. *Pediatrics International*.

[33] Zhang L., Cai K., Wang Y. (2019). The pulsatile gonadorelin pump induces earlier spermatogenesis than cyclical gonadotropin therapy in congenital hypogonadotropic hypogonadism men. *American Journal of Men’s Health*.

[34] Warne D. W., Decosterd G., Okada H., Yano Y., Koide N., Howles C. M. (2009). A combined analysis of data to identify predictive factors for spermatogenesis in men with hypogonadotropic hypogonadism treated with recombinant human follicle-stimulating hormone and human chorionic gonadotropin. *Fertility and Sterility*.

[35] Nieschlag E., Bouloux P. G., Stegmann B. J., Shankar R. R., Guan Y., Tzontcheva A. (2017). McCrary Sisk C., Behre H.M.: an open-label clinical trial to investigate the efficacy and safety of corifollitropin alfa combined with hCG in adult men with hypogonadotropic hypogonadism. *Reproductive Biology and Endocrinology*.

[36] Mitchell A. L., Dwyer A., Pitteloud N., Quinton R. (2011). Genetic basis and variable phenotypic expression of Kallmann syndrome: towards a unifying theory. *Trends in Endocrinology and Metabolism: TEM*.

[37] Men M., Wu J., Zhao Y. (2020). Genotypic and phenotypic spectra of FGFR1, FGF8, and FGF17 mutations in a Chinese cohort with idiopathic hypogonadotropic hypogonadism. *Fertility and Sterility*.

[38] Sykiotis G. P., Hoang X.-H., Avbelj M. (2010). Congenital idiopathic hypogonadotropic hypogonadism: evidence of defects in the hypothalamus, pituitary, and testes. *The Journal of Clinical Endocrinology & Metabolism*.

[39] Tsai P.-S., Moenter S. M., Postigo H. R. (2005). Targeted expression of a dominant-negative fibroblast growth factor (FGF) receptor in gonadotropin-releasing hormone (GnRH) neurons reduces FGF responsiveness and the size of GnRH neuronal population. *Molecular Endocrinology*.

