# Supplementary material for: Clinical Characteristics and Spermatogenesis in Patients with Congenital Hypogonadotropic Hypogonadism Caused by FGFR1 Mutations
Source: Int J Endocrinol. 2020 Nov 28;2020:8873532. doi: 10.1155/2020/8873532 (PMC7737440; doi:10.1155/2020/8873532)
Supplement: Supplementary Materials — Supplementary material 1 includes two supplementary tables: supplementary table 1 shows CHH-related genes included in the panel; supplementary table 2 shows the source analysis of FGFR1, PROKR2, ANOS1 gene mutations. Supplementary material 2 shows gene sequencing flow. [file 8873532.f1.zip › 8873532.f1/supplementary material 1.docx]

Supplementary Table 1. Genes Implicated in CHH

| Gene | position in Chromosome | Function |
| --- | --- | --- |
| *ANOS1* | Xp22.31 | Migration of olfactory bulb neurons and GnRH neurons |
| *FEZF1* | 7q31.32 | Important for the migration of GnRH neurons and the formation of HPG axis |
| *CCDC141* | 2q31.2 | Cytoskeleton related proteins, cortical neuron migration |
| *CHD7* | 8q12.2 | DNA unwinding, histone binding and chromosome reconstruction, GnRH and olfactory neuron formation |
| *HESX1* | 3p14.3 | Encodes a conserved homeobox protein that is a transcriptional repressor in the developing forebrain and pituitary gland |
| *FGF8* | 10q24.32 | Differentiation and migration of GnRH neurons/olfactory bulb neurons |
| *FGFR1* | 8p11.23 | Differentiation and migration of GnRH neurons / olfactory bulb neurons |
| *FLRT3* | 20p12.1 | Encodes a member of the fibronectin leucine transmembrane protein (flrt) family, which may play a role in cell adhesion and / or receptor signaling |
| *FSHB* | 11p14.1 | The pituitary glycoprotein hormone family includes follicle-stimulating hormone, luteinizing hormone, chorionic gonadotropin, and thyroid-stimulating hormone |
| *GNRH1* | 8p21.2 | Promote the secretion of LH / FSH in pituitary |
| *GNRHR* | 4q13.2 | Promote the secretion of LH / FSH in pituitary |
| *LEP* | 7q32.1 | Regulate appetite and promote GnRH secretion |
| *LEPR* | 1p31.3 | Regulate appetite and promote GnRH secretion |
| *HS6ST1* | 2q14.3 | Important for the development branch of neurons |
| *IL17RD* | 3p14.3 | FGF inhibitor, feedback regulation of Ras-MAPK signal |
| *KISS1* | 1q32.1 | Promoting GnRH secretion |
| *KISS1R* | 19p13.3 | Promoting GnRH secretion |
| *LHX4* | 1q25.2 | Involved in the regulation of pituitary differentiation and development |
| *POLR3A* | 10q22.3 | The protein encoded by this gene is the catalytic component of RNA polymerase III, which synthesizes small RNAs |
| *POLR3B* | 12q23.3 | The gene encodes the second largest subunit of RNA polymerase III, which is responsible for the synthesis of small ribosomal RNA in transfer and eukaryotes |
| *NSMF* (*NELF)* | 9q34.3 | The protein encoded by the gene is involved in olfactory axon projection and the migration of luteinizing hormone releasing hormone neurons |
| *OTX2* | 14q22.3 | The gene encodes a member of a two-family subfamily containing homologous domain transcription factors |
| *PCSK1* | 5q15 | Endonuclease, processing precursor hormone (including GnRH) |
| *PNPLA6* | 19p13.2 | Abnormal protein function leads to neurodegenerative diseases and reproductive failure |
| *PROK2* | 3p13 | Migration of neural stem cells / GnRH neurons in olfactory bulb |
| *PROKR2* | 20p12.3 | Migration of neural stem cells / GnRH neurons in olfactory bulb |
| *PROP1* | 5q35.3 | The gene encodes a pair of homologous domain transcription factors in the developing pituitary gland |
| *SEMA3A* | 7q21.11 | It is involved in the development of olfactory system and neural regulation in adolescence |
| *SEMA3E* | 7q21.11 | It is involved in the development of olfactory system and the migration of GnRH neurons |
| *SMCHD1* | 18p11.32 | This gene encodes a protein which contains a hinge region domain found in members of the SMC (structural maintenance of chromosomes) protein family |
| *WDR11* | 10q26.12 | This gene encodes a member of the WD repeat protein family |
| *LHB* | 19q13.33 | The proteins that are essential for the initiation of puberty and the Leydig are critical for the function of testicular cells |
| *SPRY4* | 5q31.3 | Determining gender differentiation |
| *TAC3* | 12q13.3 | The gene encodes a member of the rapid kinin family of secreted neuropeptides |
| *TACR3* | 4q24 | The gene belongs to a gene family and acts as a tachykinin receptor |
| *FGF17* | 8p21.3 | The gene encodes a member of the fibroblast growth factor (FGF) family. |
| *SOX10* | 22q13.1 | Transcription factors, promote the function of glial cell specific transcription factors |
| *SOX2* | 3q26.33 | This intronless gene encodes a member of the SRY-related HMG-box (SOX) family of transcription factors involved in the regulation of embryonic development and in the determination of cell fate. |
| *DMXL2* | 15q21.2 | Various neuroendocrine syndromes |

Supplementary Table 2. Source analysis of *FGFR1, PROKR2, ANOS1* gene mutations

| Mutated gene |  | De novo | Inherit from mother | Inherit from father |
| --- | --- | --- | --- | --- |
| *FGFR1* |  | 11 | 0 | 1 |
| *PROKR2* |  | 0 | 5 | 5 |
| *ANOS1* |  | 0 | 6 | 0 |
