# Supplementary material for: Clinical Characteristics and Spermatogenesis in Patients with Congenital Hypogonadotropic Hypogonadism Caused by FGFR1 Mutations
Source: Int J Endocrinol. 2020 Nov 28;2020:8873532. doi: 10.1155/2020/8873532 (PMC7737440; doi:10.1155/2020/8873532)
Supplement: Supplementary Materials — Supplementary material 1 includes two supplementary tables: supplementary table 1 shows CHH-related genes included in the panel; supplementary table 2 shows the source analysis of FGFR1, PROKR2, ANOS1 gene mutations. Supplementary material 2 shows gene sequencing flow. [file 8873532.f1.zip › 8873532.f1/supplementary material 2.docx]

***DNA Library Preparation***

Each DNA sample is quantiﬁed by agarose gel electrophoresis and Nanodrop (Thermo). Libraries were prepared using Illumina standard protocol. Briefly, 3 microgram of genomic DNA was fragmented by nebulization, the fragmented DNA is repaired, an ‘A’ is ligated to the 3′ end, Illumina adapters are then ligated to the fragments, and the sample is size selected aiming for a 350–400 base pair product. The size selected product is PCR amplified (Each sample is tagged with a unique index during this procedure), and the final product is validated using the Agilent Bioanalyzer.

***Targeted genes enrichment and sequencing***

The amplified DNA was captured with a CHH-disease related Gene Panel using biotinylated oligo-probes (MyGenostics GenCap Enrichment technologies). The capture experiment was conducted according to manufacturer’s protocol. In brief, 1μg DNA library was mixed with Buffer BL and GenCap gene panel probe (MyGenostics, Beijing, China), heated at 95°C for 7 min and 65°C for 2 min on a PCR machine; 23μl of the 65°C prewarmed Buffer HY (MyGenostics, Beijing, China) was then added to the mix, and the mixture was held at 65°C with PCR lid heat on for 22 hours for hybridization. 50 μl MyOne beads (Life Technology) was washed in 500μL 1X binding buffer for 3 times and resuspended in 80μl 1X binding buffer. 64 μl 2X binding buffer was added to the hybrid mix, and transferred to the tube with 80μl MyOne beads. The mix was rotated for 1 hour on a rotator. The beads were then washed with WB1 buffer at room temperature for 15 minutes once and WB3 buffer at 65°C for 15 minutes three times. The bound DNA was then eluted with Buffer Elute. The eluted DNA was finally amplified for 15 cycles using the following program: 98℃ for 30 s (1 cycle); 98℃ for 25 s, 65℃ for 30 s, 72 ℃ for 30 s (15 cycles); 72 ℃ for 5 min (1 cycle). The PCR product was purified using SPRI beads (Beckman Coulter) according to manufacturer’s protocol. The enrichment libraries were sequenced on Illumina HiSeq 2000 sequencer for paired read 100bp.

***Bioinformatics analysis***

After HiSeq 2000 sequencing, high-quality reads were retrieved from raw reads by filtering out the low quality reads and adaptor sequences using the Solexa QA package and the cutadapt program (<http://code.google.com/p/cutadapt/>), respectively. SOAPaligner program was then used to align the clean read sequences to the human reference genome (hg19). After the PCR duplicates were removed by the Picard software, the SNPs was firstly identified using the SOAPsnp program (<http://soap.genomics.org.cn/soapsnp.html>). Subsequently, we realigned the reads to the reference genome using BWA and identified the insertions or deletions (InDels) using the GATK program <http://www.broadinstitute.org/gsa/wiki/index.php/Home_Page>). The identified SNPs and InDels were annotated using the Exome-assistant program (<http://122.228.158.106/exomeassistant>). MagicViewer was used to view the short-read alignment and validate the candidate SNPs and InDels.
